# Improvement of the Trapezoid Method Using Raw Landsat Image Digital Count Data for Soil Moisture Estimation in the Texas (USA) High Plains

**DOI:** 10.3390/s150101925

**Published:** 2015-01-16

**Authors:** Sanaz Shafian, Stephan J. Maas

**Affiliations:** 1 Department of Plant and Soil Science, Texas Tech University, Lubbock, TX 79409, USA; 2 Department of Plant and Soil Science, Texas Tech University and Research Center, Texas A&M, Lubbock, TX 79409, USA; E-Mail: stephen.maas@ttu.edu

**Keywords:** thermal infrared, raw image digital count, soil moisture, estimation

## Abstract

Variations in soil moisture strongly affect surface energy balances, regional runoff, land erosion and vegetation productivity (*i.e.*, potential crop yield). Hence, the estimation of soil moisture is very valuable in the social, economic, humanitarian (food security) and environmental segments of society. Extensive efforts to exploit the potential of remotely sensed observations to help quantify this complex variable are ongoing. This study aims at developing a new index, the Thermal Ground cover Moisture Index (TGMI), for estimating soil moisture content. This index is based on empirical parameterization of the relationship between raw image digital count (DC) data in the thermal infrared spectral band and ground cover (determined from raw image digital count data in the red and near-infrared spectral bands).The index uses satellite-derived information only, and the potential for its operational application is therefore great. This study was conducted in 18 commercial agricultural fields near Lubbock, TX (USA). Soil moisture was measured in these fields over two years and statistically compared to corresponding values of TGMI determined from Landsat image data. Results indicate statistically significant correlations between TGMI and field measurements of soil moisture (R^2^ = 0.73, RMSE = 0.05, MBE = 0.17 and AAE = 0.049), suggesting that soil moisture can be estimated using this index. It was further demonstrated that maps of TGMI developed from Landsat imagery could be constructed to show the relative spatial distribution of soil moisture across a region.

## 1. Introduction

Soil moisture is a key factor in controlling the exchange of water and heat energy flux between the land surface and atmosphere through evaporation and transpiration processes [1–3]. Information on the distributed soil moisture at larger scales with sufficient spatial and temporal resolution is needed for improving climatic and hydrologic modeling and prediction [4]. In addition, information on soil moisture is of great use in crop management, including irrigation scheduling. Early detection of dry conditions is important for yield forecasting, which can serve as an early warning system in agriculture.

Various approaches have been developed to estimate soil moisture, from ground-based sampling e.g., [5,6] to air/space-borne remote sensing techniques e.g., [3,7–15]. Ground-based methods involve point measurements, so local scale variations in soil properties, terrain, and vegetation cover make the selection of representative field sites difficult, if not impossible [3,16]. Moreover, field methods are complex, labor intensive and expensive. Therefore, *in situ* measurements may not adequately represent the spatial distribution of soil moisture content and are not available for continuous spatial and temporal coverage at regional and global scales. In contrast, remote sensing (RS) techniques are promising because they produce spatially explicit measurements [17]. For large areas, the cost of acquiring RS data may be less than ground-based methods. Since the 1970s, a number of remote sensing methods have been developed to quantify soil moisture using different regions of electromagnetic spectrum, from the optical to microwave regions [8,11,14,18–22].

A wide variety of models for estimating soil moisture have been developed using various satellite data. They are typically based on satellite-derived Vegetation Indices (VIs) evaluated from visible and near infrared data and/or surface temperature (T_s_) estimated from thermal band data. The applications of approaches combining VIs and T_s_ dates back to the 70's and are typically based on the concept for detecting canopy water stress or crop evapotranspiration [23]. Indices such as the Crop Water Stress Index (CWSI) were developed to be used for irrigation scheduling [24]. Over the past 40 years, the T_s_–VI concept has been used in various applications, such as the estimation of soil moisture and evapotranspiration. A number of studies have documented the T_s_–VI relationship and have described a geometric (triangular or trapezoidal) representation of the data falling between the T_s_ and the VI axes [11,12,20,25–30]. The application of the T_s_–VI concept for soil moisture content estimation began with the work of Nemani *et al.* [31], who took advantage of the spatial information offered by satellite data to infer canopy conductance from the slope of the T_s_–VI relationship. They found a strong negative correlation between T_s_ and VI expressed as the Normalized Difference Vegetation Index (NDVI) for all biome types. The concept was further developed by Moran *et al.* [14], who used the trapezoidal shape of NDVI *versus* (T_s_–T_air_) to estimate plant canopy water stress. This “VIT trapezoid” was an attempt to combine spectral vegetation indices with composite surface temperature measurements to evaluate evapotranspiration rates for sites with full or partial vegetation cover [14]. At about the same time, Carlson *et al.* [22] proposed a universal triangular method to explore the relationship between soil moisture availability, T_s_ and NDVI. Since then, trapezoid or triangle relationships between VI and T_s_ have been widely studied to estimate soil moisture and energy flux at different spatial scales using RS data from different sources [32,33].

All of the previously discussed methods require converting thermal remote sensing to surface temperature data. This conversion is time-consuming and requires the collection of additional information, which can be expensive. In addition, small errors in computed surface temperature can lead to unreasonable values of the surface energy fluxes [32]. A drawback of the triangle or trapezoid approaches is that establishing the “dry edge” of the T_s_–VI space is often not straightforward [11] http://www.sciencedirect.com.lib-e2.lib.ttu.edu/science/article/pii/S0034425701002747 - BIB32, because different surface types can have different slopes and intercepts for the dry edge even under equal atmospheric and surface moisture conditions. A number of studies have been conducted to identify the dry edge and estimate its slope and intercept. Some of these efforts have had only theoretical bases [14], some have been based on *in situ* measurements [34]http://www.sciencedirect.com.lib-e2.lib.ttu.edu/science/article/pii/S0034425701002747 - BIB10, and others have been based largely on finding a “best fit” to the dry edge as it varies from image to image [11,20,29,32]. The objective of this study is to present a simple method for estimating soil moisture in agricultural regions using raw remote sensing data without calibration or conversion to surface reflectance or temperature. This method represents modification of the approach proposed by Moran *et al.* [14] consisting of replacing NDVI by vegetation ground cover (*GC*) and T_s_–T_air_ by raw thermal digital counts (*TIRDC*). The method is conceptually and computationally straightforward, and only satellite-derived information is needed. The ability of the method to estimate soil moisture is tested using independent measurements of soil moisture obtained from agricultural fields in the study region. We also demonstrate the ability of the method to map variations in soil moisture across an agricultural region.

## 2. Materials and Methods

### 2.1. Conceptual Basis

As shown by numerous investigators, plotting values of VI *versus* corresponding values of T_s_ derived from multispectral satellite imagery produces the characteristic “triangle” or “trapezoid” distribution [11,14,20,25,29]. Vegetation indices such as NDVI are indicators of the amount of vegetation in the scene. However, they are not direct measures of the amount of vegetation and are usually related to measures of vegetation density (such as *GC* or LAI) empirically. In an earlier study, Carlson [32] replaced VI with fractional vegetation cover and showed that this replacement does not change the shape of the triangular or trapezoidal distribution. Carlson [32] used fractional vegetation cover instead of VI in an attempt to establish a more universal triangle for estimating soil moisture availability. Some authors [35] favor a linear relationship between NDVI and fractional vegetation cover, rather than the equation proposed by Carlson [32]. Maas and Rajan [36] demonstrated that fractional vegetation cover can be directly evaluated from satellite image DC data in the red and near-infrared spectral bands and they called it vegetation ground cover (*GC*). We propose replacing VI in the triangular or trapezoidal distributions with remotely sensed *GC*. The advantage of this method is that *GC* can be calculated directly from raw satellite image DC data. In addition, *GC* provides a more direct interpretation of the interaction of plant canopy density and plant canopy temperature.

**Figure 1.**
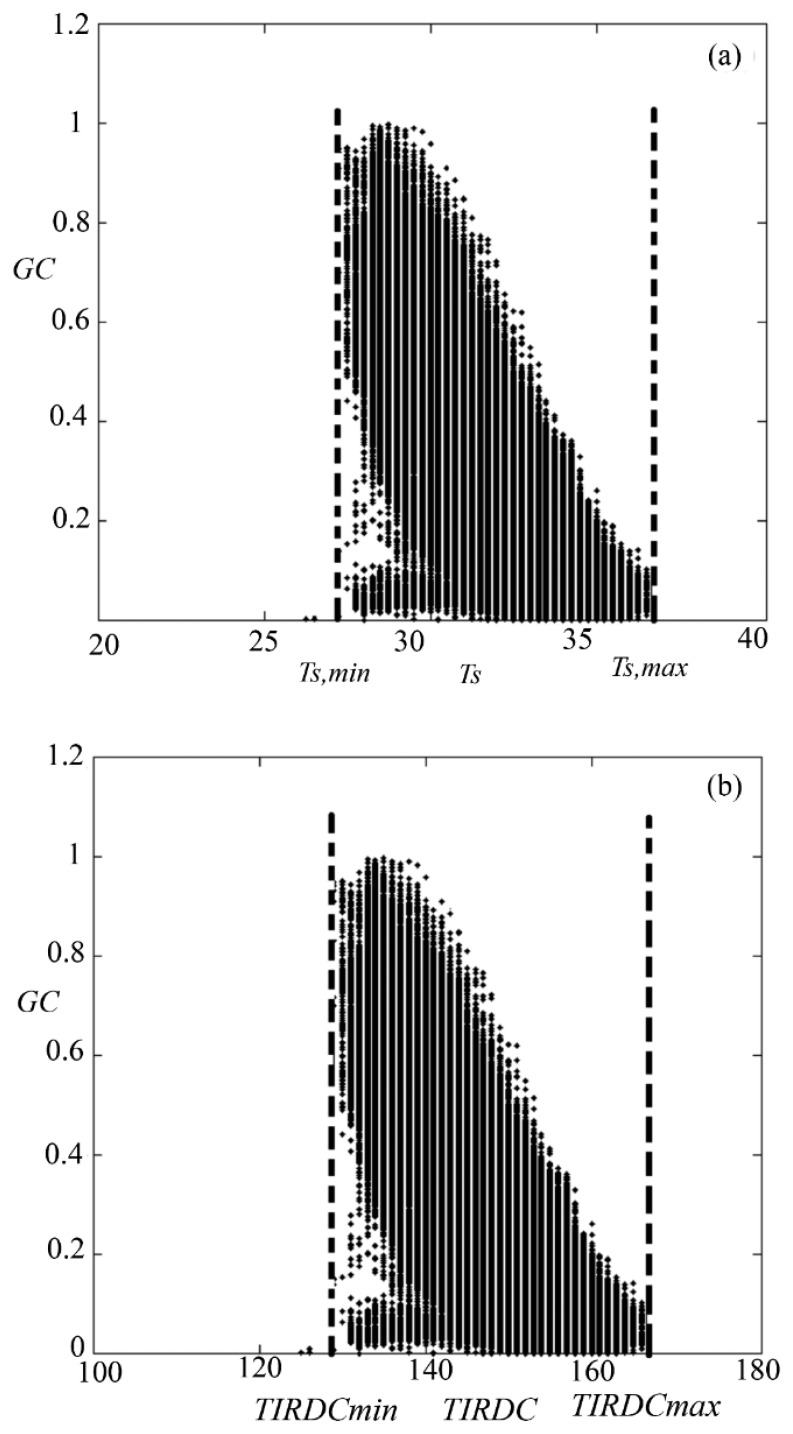
Result of plotting vegetation *GC* (result of plotting DC in Red and NIR spectral bands of Lnadsat-7) *versus* (**a**) surface temperature T_s_; (**b**) raw thermal infrared digital count data (*TIRDC*) for pixels comprising a medium-resolution multispectral satellite image (Landsat-7) of an agricultural region.

Figure 1a shows the typical distribution of points resulting from plotting values of vegetation *GC* (result of plotting Digital Count (DC) in Red and NIR spectral bands) *versus* corresponding values of surface temperature (T_s_) for pixels comprising a medium-resolution multispectral satellite image (Landsat-7) of an agricultural region. Figure 1b shows a similar distribution, but in this case T_s_ has been plotted *versus* the raw thermal infrared digital count values (*TIRDC*) of Landsat-7 used in calculating T_s_. When properly scaled, the shapes of the distributions in the two figures are the same. The advantage of working with the distribution in Figure 1b is that it can be constructed from raw satellite DC data without the need for atmospheric or radiometric calibration (recall that *GC* can be determined directly from raw DC data as described in the previous paragraph).

**Figure 2.**
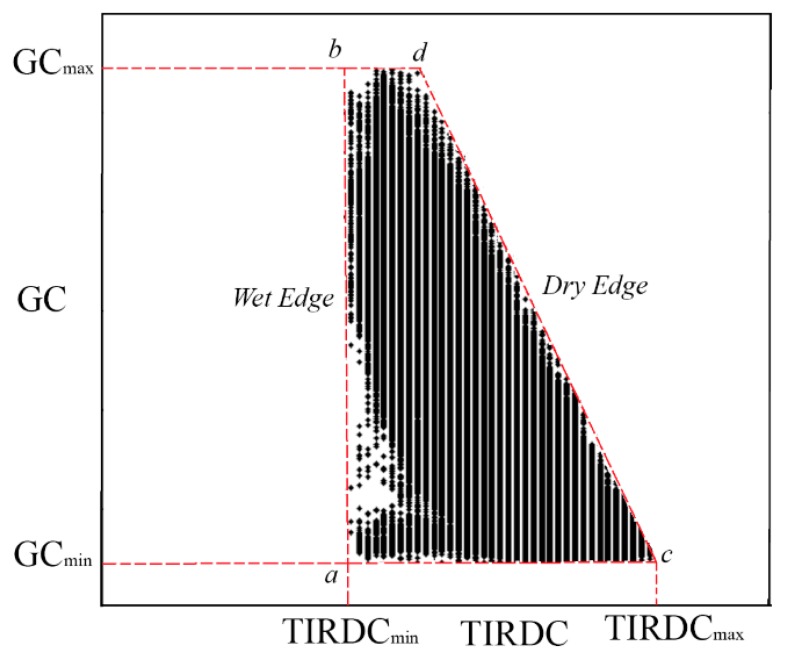
Diagrammatic representation of the distribution of vegetation *GC versus* raw thermal infrared digital count data (*TIRDC*) like that presented in Figure 1b.

The distribution shown in Figure 1b can be described diagrammatically by Figure 2. Points along the left edge of the distribution (line connecting points *a* and *b*) represent pixels with relatively cool surface temperatures, either from high evaporation rates from the wet soil surface under low *GC* conditions (near point *a*), or from high transpiration rates from the vegetation canopy under high *GC* conditions associated with high soil moisture contents and a lack of water stress (near point *b*). A combination of both of these effects may be present at intermediate *GC* levels. In contrast, points along the right edge of the distribution (line connecting points *c* and *d*) represent pixels with relatively warm surface temperatures, either from low evaporation rates from the dry soil surface under low *GC* conditions (near point *c*), or from low transpiration rates from the vegetation canopy under high *GC* conditions associated with low soil moisture contents leading to stomatal closure in the canopy (near point *d*). Again, a combination of both of these effects may be present at intermediate *GC* levels. Since the left edge of the distribution is generally associated with wetter soil moisture conditions, it is often called the “wet edge”. The “wet edge” represents the situation where there is enough water to allow evaporation to occur at unrestricted rates and the vegetation is not stressed by the lack of soil moisture. This line corresponds to the wet edge of the temperature–vegetation dryness index (TVDI) proposed by Sandholt [11].

The right edge of the distribution, which is generally associated with drier soil moisture conditions, is often called the “dry edge.” The dry edge represents the maximum soil water-limiting conditions for the plant canopy [37]. The position of a point between the wet and dry edges of the trapezoid is indicative of its soil moisture content. Note that it is the position of the point relative to the wet and dry edges, and not the absolute value of the point, that is important in indicating its soil moisture content. Thus, plotting *GC versus TIRDC* (Figure 1b) provides similar information as plotting *GC versus* T_s_ (Figure 1a), since both distributions of points contain the characteristic wet and dry edges. Values of *GC* in Figure 2 will range from 0 (bare soil) to 1 (full canopy). Values of *TIRDC* between image acquisitions may vary due to differences in weather conditions, surface conditions, and atmospheric clarity. Normalizing *TIRDC* by its maximum and minimum values within the distribution can remove this image-to-image variation. Normalizing the *TIRDC* distribution results in both coordinate axes varying from 0 to 1 regardless of the amount of net radiation or the ambient air temperature and thermal radiation [32]. Normalization of *TIRDC* can be accomplished using the following equation,

(1) $$\text{TIRD}C_{\text{norm},i}=\left(\text{TIRD}C_{i}-\text{TIRD}C_{\text{min}}\right)/\left(\text{TIRD}C_{\max}-\text{TIRD}C_{\text{min}}\right)$$

in which *TIRDC_norm,i_* is the normalized value of *TIRDC* for a given pixel, *TIRDC_max_* is the maximum value of *TIRDC* representing dry, bare soil (point *c* in Figure 2), and *TIRDC_min_* is the minimum value of *TIRDC* representing non-stressed full vegetation canopy (point *b* in Figure 2).

#### 2.1.1. *TIRDC_norm_*–*GC* Space

**Figure 3.**
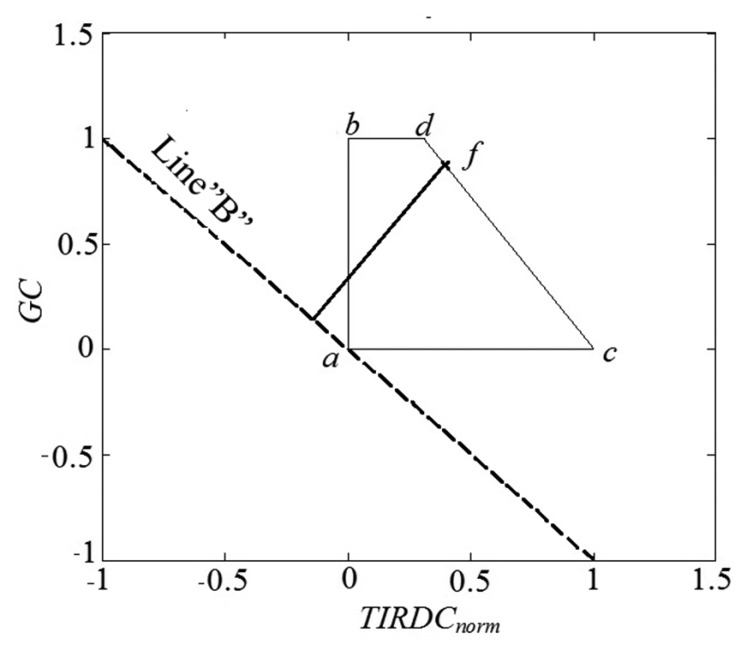
*TIRDC_norm_*–*GC* space used for determining the vertex *d* of the trapezoid. Line “B” passes through point *a* with a slope of −1 and serves as a baseline for measuring perpendicular distance across the *TIRDC_norm_*–*GC* space. In this example, point *f* is the point in the distribution of observed pixel values that has the maximum perpendicular distance from line “B”.

*TIRDC_norm_*–*GC* distributions fit within a trapezoidal space (Figure 3) that has three fixed vertices: point *a* (*TIRDC_norm_* = 0, *GC* = 0), point *b* (*TIRDC_norm_* = 0, *GC* = 1), and point *c* (*TIRDC_norm_* = 1, *GC* = 0). Since the dry edge of the distribution likely represents the driest soil moisture conditions over the imaged scene, the gradient in soil moisture within the scene should be roughly perpendicular to the orientation of the dry edge. Thus, it is important to know the position of the dry edge. Since point *c* is fixed in the *TIRDC_norm_*–*GC* space, this comes down to determining the location of point *d*. The position of point *d* usually cannot be determined directly from the distribution of observed pixel data, since it is uncommon to find vegetation that is severely stressed yet has *GC* = 1. Figure 3 shows a method to estimate the location of point *d* and thereby establish the location of the dry edge. A straight line with a slope of −1 (dashed line “B” in Figure 3) is placed through point *a*. The choice of a slope of −1 is somewhat arbitrary but should place the orientation of line “B” roughly perpendicular to the soil moisture gradient. This line is used as a baseline for measuring distance along the gradient. The observed point in the *TIRDC_norm_*–*GC* distribution that has greatest perpendicular distance from the baseline “B” is found by inspection (indicated by point *f* in Figure 3). Point *d* can be considered to be the point where a straight line from point *c* passing through point *f* intersects the top of the trapezoid (*GC* = 1). It has been our experience that, for agricultural regions with a mixture of field crops, perennial pastures, and natural vegetation, the distribution of image pixel values will usually allow identification of point *f* during most of the growing season. However, the authors recognize that the approach in the form described in this article might not be directly applicable to all situations.

Knowing the positions of points *c* and *d*, the equation of the dry edge can be written as follows:

(2) $$GC_{\text{i}}=\frac{GC_{d}}{\text{TIRD}C_{\text{norm},d}-1}\times \text{TIRD}C_{\text{norm},\max,i}-\frac{GC_{d}}{\text{TIRD}C_{\text{norm},d}-1}$$

where *GC_i_* is the value of *GC* for a given image pixel at the dry edge, *TIRDC_norm,max,i_* is the value of normalized *TIRDC* at that pixel, and *GC_d_* and *TIRDC_norm,d_* are the *GC* and normalized *TIRDC* values, respectively, observed for point *d*. By rewriting Equation (2), the value of *TIRDC_norm,max,i_* can be calculated for any point along the dry edge:

(3) $$\text{TIRD}C_{\text{norm},\max,i}=\left(GC_{i}+\frac{GC_{d}}{\text{TIRD}C_{\text{norm},d}-1}\right)/\left(\frac{GC_{d}}{\text{TIRD}C_{\text{norm},d}-1}\right)$$

#### 2.1.2. Thermal Ground Cover Moisture Index

**Figure 4.**
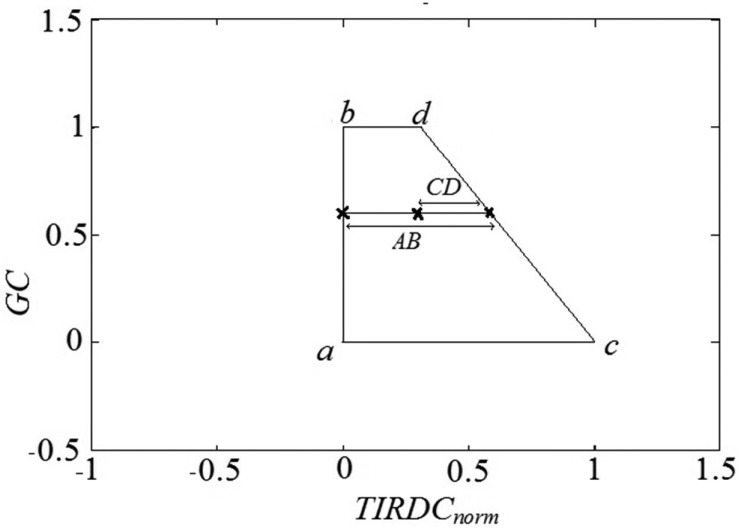
An illustration of the *TIRDC_norm_*–*GC* space used for determining Thermal Ground Cover Moisture Index (*TGMI*). For a given pixel, CD and AB are used to calculate the *TGMI*.

An index, the Thermal Ground cover Moisture Index (*TGMI*), can be defined based on the relative position of a point in the *TIRDC_norm_*–*GC* space depicted in Figure 3. The *TGMI* is similar to the WDI described by Moran *et al.* [14] but can be evaluated from raw image DC data. *TGMI* is shown diagrammatically in Figure 4. The value of *TGMI* for a given image pixel can be evaluated as follows:

(4) $$\text{TGM}I_{\text{i}}=\frac{CD}{AB}=\frac{\text{TIRD}C_{\text{norm},\max,i}-\text{TIRD}C_{\text{norm},i}}{\text{TIRD}C_{\text{norm},\max,i}-\text{TIRD}C_{\text{norm},\text{min}}}$$

where TGMI_i_ is the value of *TGMI* calculated for a point within the *TIRDC_norm_*–*GC* space, *TIRDC_norm,min_* is the minimum normalized *TIRDC* value at the wet edge (equal to 0), *TIRDC_norm,i_* is the observed normalized *TIRDC* value at given pixel image and *TIRDC_norm,max,i_* defines the maximum normalized *TIRDC* value at the dry edge calculated from Equation (3). *TGMI* has the values of 1 at the “wet edge” and 0 at the “dry edge.” Now by substituting Equation (3) into Equation (4), *TGMI* can be rewritten as follows:

(5) $$\text{TGM}I_{\text{i}}=1-\frac{\text{TIRD}C_{\text{norm},i}\times GC_{\text{d}}}{\left(\text{TIRD}C_{\text{norm},d}-1\right)\times GC_{i}+GC_{d}}$$

where *GC_i_* is the *GC* value for given pixel image, and *GC_d_* and *TIRDC_norm,d_* are the *GC* value and normalized *TIRDC* value at point *d*, respectively.

The value of *TGMI* should be proportional to the volumetric soil water content (VWC) present at the site of a given pixel. TGMI calculated in Equation (5) changes between 0 and 1, while soil moisture content varies between 0 (at dry edge) and soil moisture content at saturation (at wet edge) that depends on the soil texture and characteristics. Considering the range of soil moisture content at dry soil and saturated soil, TGMI can be normalized between 0 and soil moisture content at the saturation (*VWC_s_*) using Equation (6). The result of this equation represents soil moisture content for each pixel:

(6) $${\text{VWC}}_{i}=\text{TGMIi}\times {\mathrm{VWC}}_{s}$$

### 2.2. Field Study

**Figure 5.**
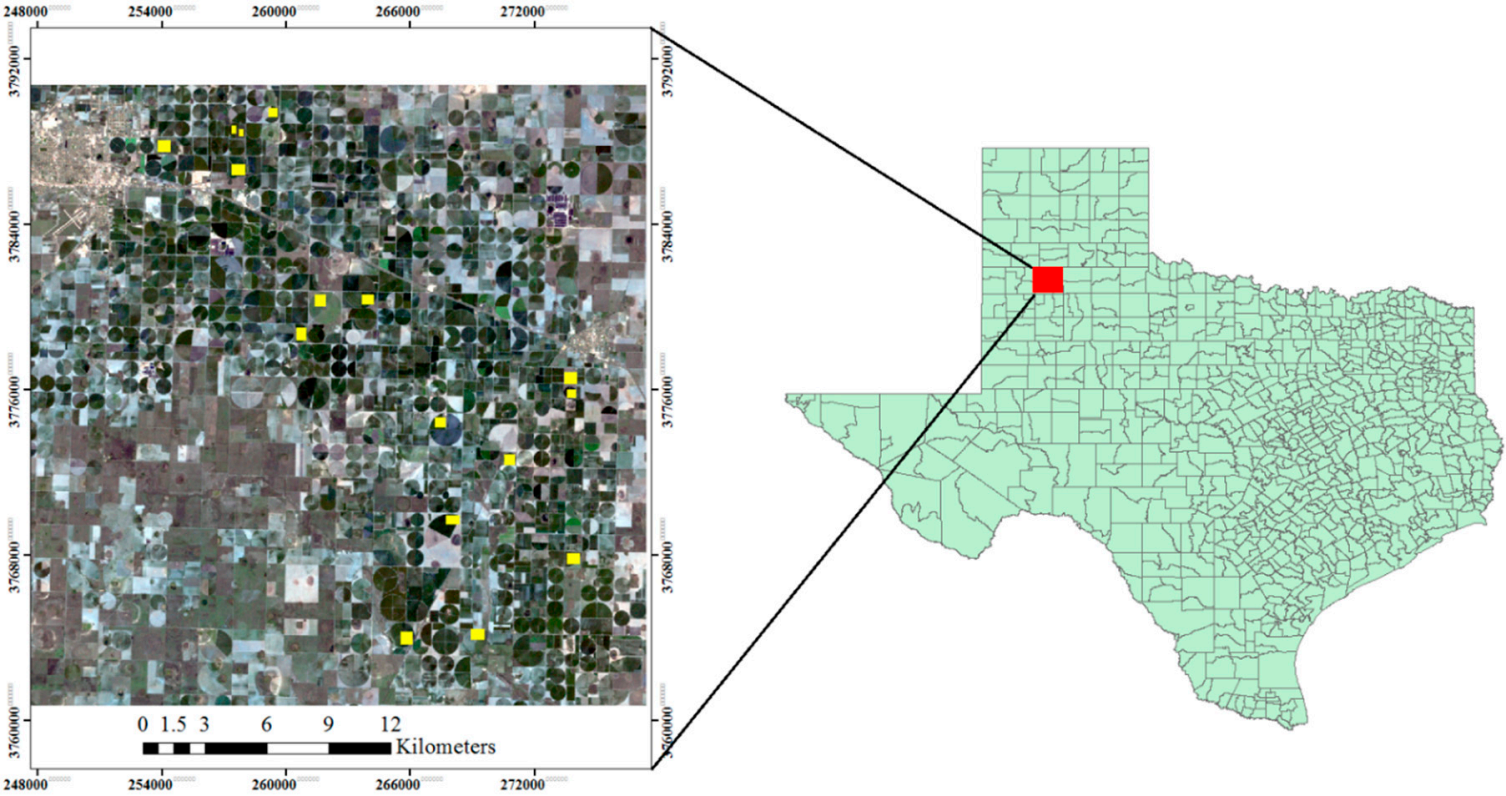
Map and experiment stations in the study area.

Performance of the *TGMI* approach under different environmental conditions was evaluated using data from 18 commercial fields in the Southern High Plains of Texas (Figure 5). The fields used in this study were part of the Texas Alliance for Water Conservation (TAWC) Demonstration Project, a large project conducted in this region to promote conservation of regional water resources. Predominant soils in the study area are non-calcareous clay loams and loams in the Pullman and Pullman-Olton associations [38] with general value of 0.5 for *VWC_s_* [38].

The study involved the acquisition and analysis of multispectral satellite imagery for calculating *TGMI* and measurement of volumetric soil water content for comparison with the corresponding calculated *TGMI* values. Methods of data collection and analysis are described in the following sections.

### 2.3. Satellite Image Data

Satellite data for calculating *TGMI* were obtained from Landsat imagery. In 2012, Landsat-7 Enhanced Thematic Mapper (ETM) imagery containing the study site was acquired on 5 dates spanning the period when soil moisture sensors were installed in study fields. These acquisitions were supplemented by Landsat-8 Operational Land Imager (OLI) and Thermal Infrared Sensor (TIRS) imagery acquired on five additional dates during the 2013 growing season. Data acquisition dates are listed in Table 1. Images used in this study were numbered from 1 to 10 for easier referral. Level 1T of each image, located according to the Landsat World Reference System (WRS-2) along Path 30 at Row 36, was obtained from the U.S. Geological Survey (USGS) Earth Explorer website [39]. Pixel size in the imagery was 30 m in the visible and short wave infrared spectral bands. For the thermal infrared imagery, imagery was acquired at a lower spatial resolution (60 m for Landsat-7 and 100 m for Landsat-8) and was re-sampled to 30 m. Level 1T images provide systematic radiometric and geometric accuracy by incorporating ground control points while employing a Digital Elevation Model (DEM) for topographic accuracy [40]. A cloud mask based on simple thresholding in the visible and thermal channels was applied to all images, leaving only cloud-free pixels for our analyses.

Data extracted in the red and near-infrared spectral bands of the Landsat imagery were used to estimate *GC* for each field using the procedure described by Maas and Rajan [36]. In this procedure, a scatterplot is constructed for each image by plotting pixel DC values in the near-infrared spectral band *versus* corresponding DC values in the red spectral band. The bare soil line is identified in each scatterplot by visual inspection, allowing the value of the Perpendicular Vegetation Index (PVI) to be calculated for each image pixel [41]. The point in each scatterplot corresponding to 100% *GC* are also identified by visual inspection, and its PVI value is determined. The average PVI value for each field in the study was determined from the PVI values for the image pixels contained within the boundaries of the field. The *GC* for each field was then calculated by dividing the average PVI value by the appropriate value of PVI corresponding to 100% *GC*. These image analysis operations were performed using ENVI image processing software (ITT, Boulder, CO, USA) and MATLAB programming software (MathWorks, Natick, MA, USA).

A scatterplot was constructed for each image by plotting calculated *GC* values *versus* corresponding pixel DC values in the thermal infrared spectral band. In addition, T_s_ image were created for each image acquisition date [42] and a scatterplot was constructed for each image by plotting *GC* values *versus* corresponding pixel DC values in T_s_. The maximum value of *TIRDC* at *GC* = 0 was identified in the scatterplot, along with the minimum value of *TIRDC* at *GC* = 1. These values were used in normalizing the thermal infrared DC values according to Equation (1). The same method was used to identify “T_s,max_” and “T_s_,_min_” values. Then T_s_ values were normalized between 0 and 1. In addition, the value of *TIRDC_norm,d_* and *GC_d_* were determined for point *d* using Equation (3). Finally, *TGMI_i_* was calculated for each pixel using Equations (4) and (5). The average *TGMI* for each field in the study was determined from the *TGMI_i_* values for the image pixels contained within the boundaries of the field. These image analysis operations were performed using ENVI image processing software and MATLAB programming software.

### 2.4. Soil Moisture Data

In situ measurements of volumetric soil water content (VWC) were made for the 18 fields in the study. In 10 of the fields, we installed Model CS616 time domain reflectometry (TDR) probes (Campbell Scientific, Logan, UT, USA) at the start of the study. These were installed to measure the water content of the soil in a layer approximately 5 cm below the surface. Data were continuously recorded using either CR10X or CR1000 data loggers (Campbell Scientific). The data logger program used to read the probes utilized a factory calibration for a mineral soil to calculate volumetric soil water content from the measured dielectric constant. In an eight additional fields, volumetric soil water content was measured with commercially available capacitance probes installed by two companies as part of the TAWC project. These were either John Deere Field Connect soil moisture probes (John Deere, Moline, MO, USA) or AquaSpy soil moisture probes (AquaSpy, San Diego, CA, USA). Both systems measure soil moisture at various depths in the soil down to 150 cm. For this study, soil moisture measurements in the upper portion of the soil profile roughly corresponding to the soil layer in which the CS616 TDR probes were installed were used. Data from these probes was accessed from websites set up to monitor soil moisture in the fields as part of the TAWC project. Measurements of volumetric soil water content were extracted from the data records for each field that corresponded to the dates and times of the satellite image acquisitions (Table 1). For the CS616 probes, these data were extracted from the data logger records. For the John Deere and AquaSpy probes, these data were accessed and extracted from their respective websites.

### 2.5. Statistical Analysis

Values of VWC were calculated from *TGMI* using Equation (6) for comparison with corresponding measurements of VWC for days with satellite imagery acquisitions. In these calculations, the value of *VWC_s_* was set equal to 0.5. A paired *t*-test was used to determine if the average of the calculated VWC values were significantly different from the average of the observed VWC values of soil volumetric water content from the field measurements.

Calculated values of VWC were plotted *versus* corresponding measured soil volumetric water content. The distribution of points was fit using simple linear regression analysis. Student's *t*-tests were used to determine if the slope of the regression was significantly different from 1, and if the intercept of the regression was significantly different from zero. Were this the case, one could conclude that the regression was not significantly different from the 1:1 line, and that the *TGMI* × *VWCs* approach did a reasonably good job of estimating volumetric water content for the study. The difference was determined for each pair of calculated and measured VWC and used to calculate the Average Absolute Error (AAE) according to the equation:

(7) $$\text{AAE}=\frac{\sum_{i=1}^{n}\left|\text{TGMI}*VWC_{S}-{\text{VWC}}_{\text{measured}}\right|}{n}$$

where *n* is the number of observations. AAE can be considered as a measure of the overall accuracy of the estimation approach.

## 3. Results and Discussion

### 3.1. TIRDC–GC Space

**Figure 6.**
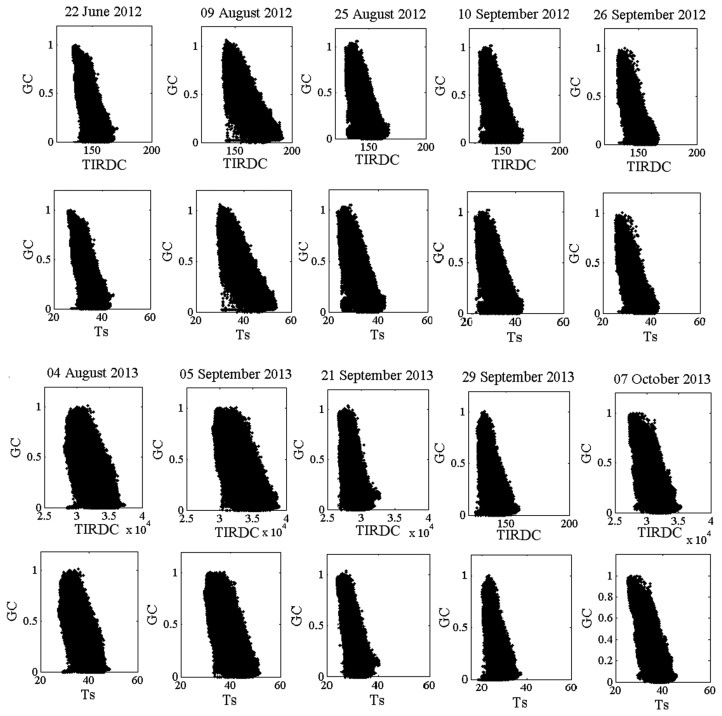
Plots of *GC* as functions of either *TIRDC* and T_s_ for each Landsat image acquisition.

**Figure 7.**
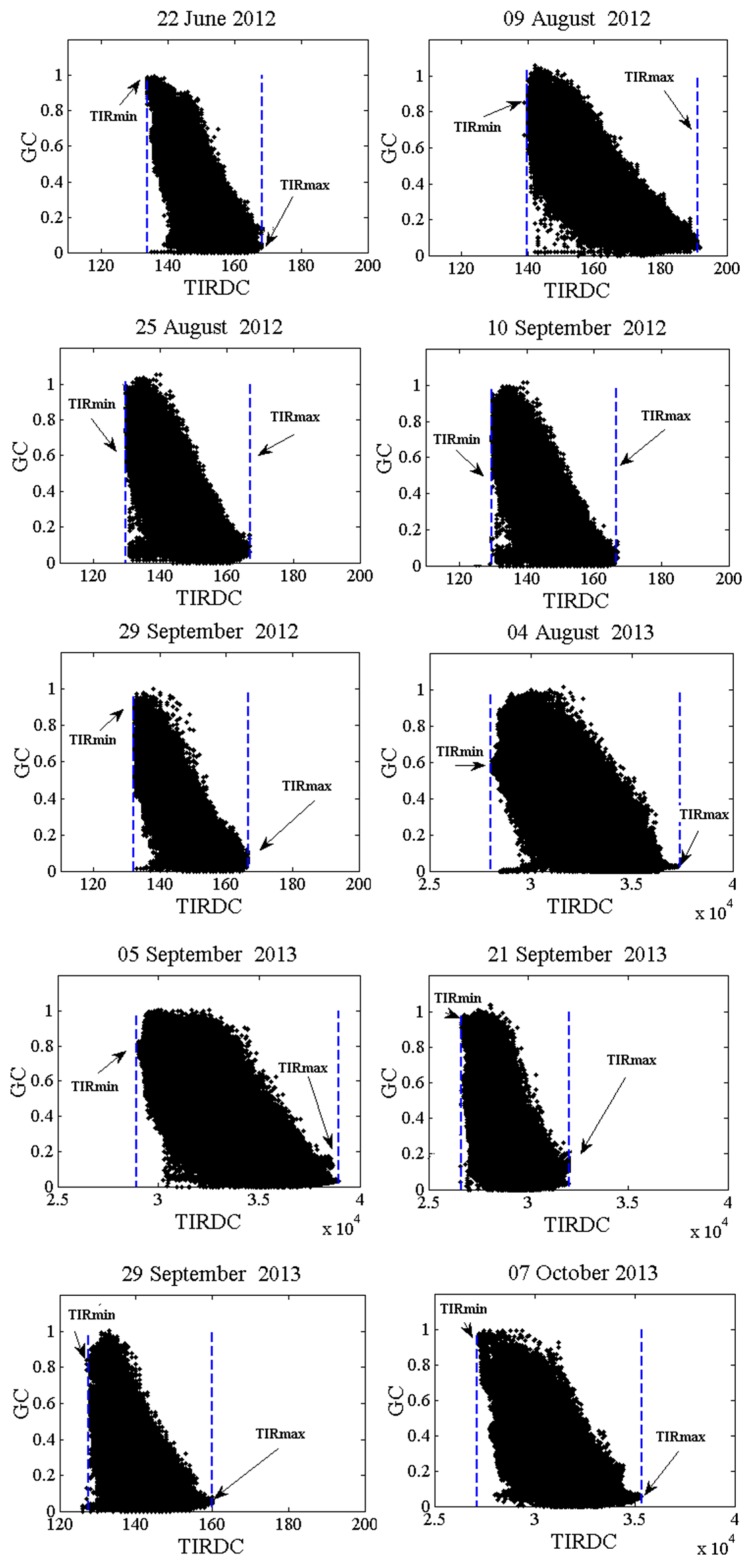
Identification of *TIRDC_max_* and *TIRDC_min_* used to normalized *TIRDC* values in the *TIRDC-GC* scatterplot.

Plotting *TIRDC* as a function of *GC* for each image showed that the trapezoidal *TIRDC*–*GC* space was well defined in all cases (see Figure 6). Figure 6 also shows that when properly scaled, the *TIRDC-GC* space has the same shape as the T_s_–*GC* space. Differences in the range of *TIRDC* values can largely be attributing to differences in net radiation, atmospheric conditions, or soil moisture conditions on the date of image acquisition. Figure 7 shows that how the points corresponding to *TIRDC_max_* and *TIRDC_min_* were defined for each *TIRDC*–*GC* scatterplot. This figure shows that *TIRDC_max_* and *TIRDC_min_* are well-defined for all cases. These values were used to normalize *TIRDC* values according to Equation (1).

### 3.2. TIRDC_norm_ Versus T_s,norm_

**Figure 8.**
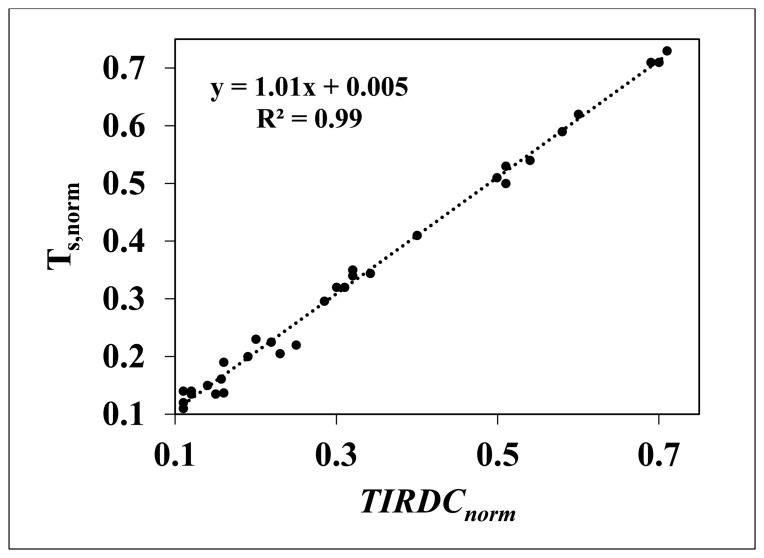
Simple linear regression between *TIRDC_norm_* and T_s_,_norm_.

A regression analysis was used to compare *TIRDC_norm_ versus* T_s,norm_. To perform this analysis, corresponding areas were selected in *TIRDC_norm_* and T_s,norm_ images constructed for each image acquisition date. Average values of *TIRDC_norm_* and T_s,norm_ for each selected area were calculated using the Region of Interest (ROI) tool in ENVI. The average values of *TIRDC_norm_* were plotted *versus* the corresponding average values of T_s_, as shown in Figure 8.

The points in this graph tend to lie along the 1:1 line. The slope and intercept of the least-square linear regression fit to these data is 1.01 and 0.005 respectively. Analysis using the Student's *t*-test indicated that the slope and intercept was not significantly different from 1 (*t* = 0.338, 31 df, α = 0.05) and the intercept was not significantly different from 0 (*t* = 0.107, 31 df, α = 0.05). Thus, the regression line through these points is not significantly different from the 1:1 line. A Student's *t*-test of the average *TIRDC_norm_* and T_s_,_norm_ values indicated that these two values are not significantly different (*t* = −0.263, 64 df, α = 0.05). From this analysis we conclude that *TIRDC_norm_* can be used to estimate soil moisture in place of T_s_,_norm_ in *TIRDC*–*GC* space.

### 3.3. TIRDC_norm_–GC Scatterplot

**Figure 9.**
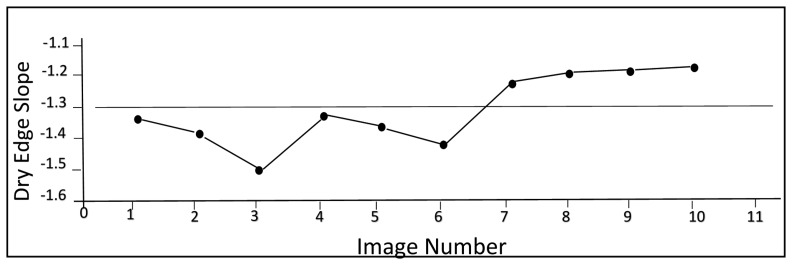
Dry edge slopes for the 10 images used in the study.

**Figure 10.**
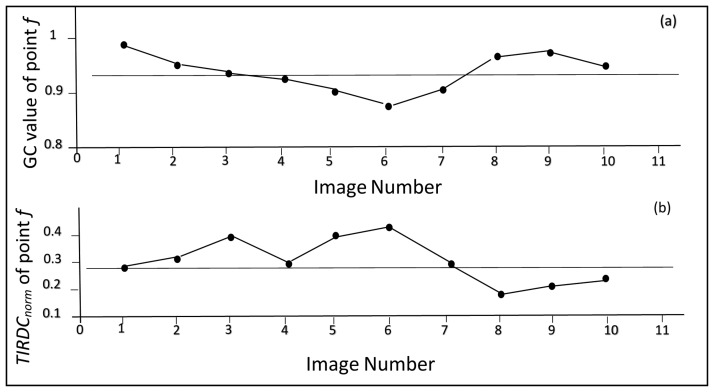
Position of point *f* in the 10 images used in the study, (**a**) *GC* value of point *f*; (**b**) *TIRDC_norm_* value of point *f*.

In order to determine the parameters describing the “dry edge”, the position of point *f* (the point corresponding to the greatest perpendicular distance from line “B” in the *TIRDC_norm_*–*GC* space) was identified for each image acquisition date using Equations (2) and (3). The slopes of the dry edge determined for the 10 images is plotted as a function of image number in Figure 9. The small variation in dry edge slopes in Figure 9 (approximately 11% of the mean slope) suggests that there was reasonable consistency in the positions of point *f* determined for the images used in the study. Figure 10a shows the *GC* value associated with each point *f* plotted *versus* image number, while Figure 10b shows the *TIRDC_norm_* values associated with it. The small variations in these values (approximately 4% of the mean *GC* value and 20% of the mean *TIRDC_norm_* value) also emphasizes the consistency among the values determined for point *f*.

**Figure 11.**
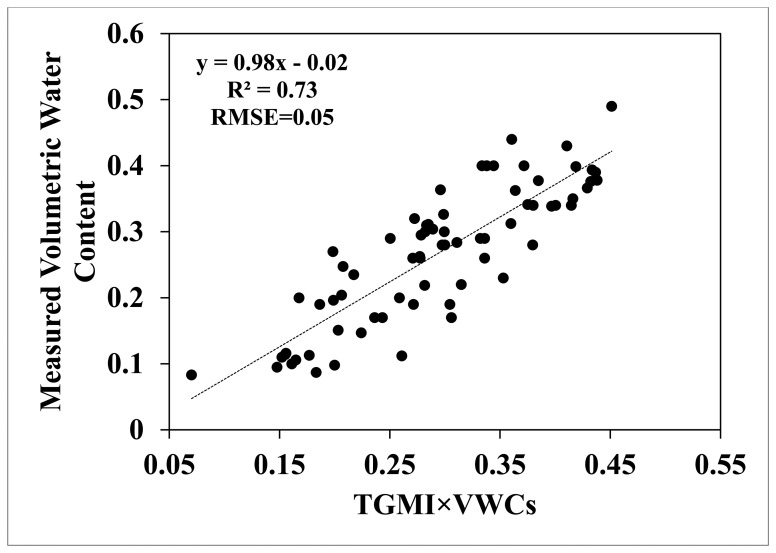
Simple linear regression between field measurements of volumetric soil water content and corresponding values of *TGMI* × VWCs calculated from multispectral satellite image data.

Figure 11 shows values of volumetric soil water content from field measurements in the 18 study fields plotted *versus* corresponding values of *TGMI* × VWC_s_ calculated from multispectral satellite image data using Equation (6). The diagonal dashed line in the figure represents the 1:1 line. The dashed line represents the simple linear regression fit to the points in the figure. This regression line has a slope of 0.98 and a y-intercept of −0.02, and explains approximately 73% of the total variance in the data with an RMSE of 0.05 and Mean Bias Error (MBE) of 0.17. The *t*-test performed to determine if the regression slope was significantly different from 1 resulted in *t* = −0.23. This value was less than the corresponding critical value (*t*_α_ = 1.99, 69 df, α = 0.05), which suggests that there was no significant difference between the slope of the regression and 1. The *t*-test performed to determine if the regression intercept was significantly different from zero resulted in *t* = −0.98 with 69 df. This value was less than the corresponding critical value (*t*_α_ = 1.99, 69 df, α = 0.05), which suggests that there was no significant difference between the y-intercept of the regression and zero. Overall, these results suggest that there was no significant difference between the regression line and the 1:1 line in this study. Thus, we conclude that the *TGMI* method was able to reasonably estimate volumetric water content in this study. The average absolute error (AAE) between *TGMI* × VWCs and measured volumetric water content values was 0.049.

**Figure 12.**
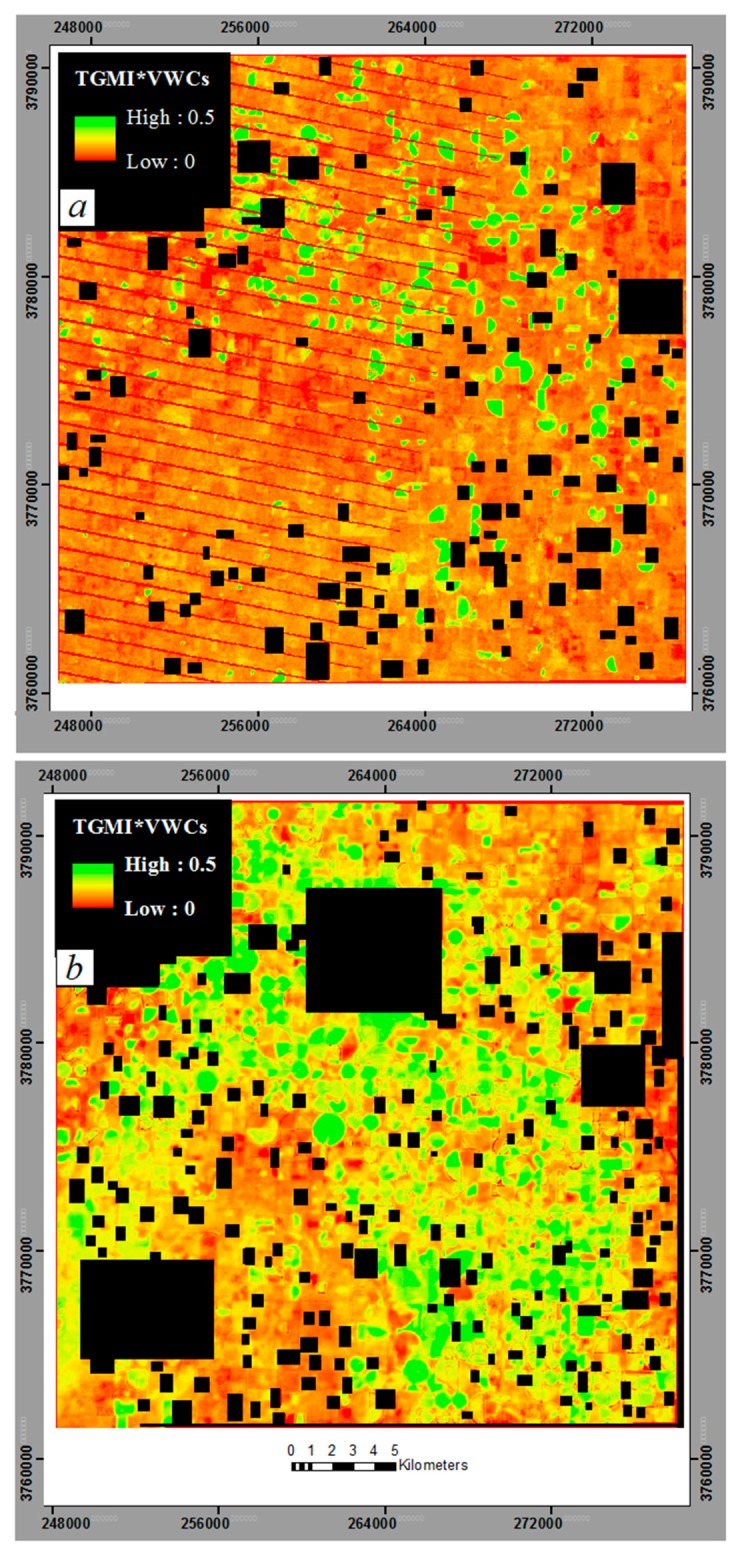
Simple *TGMI* × VWCs maps for two dates for a portion of the Texas High Plains; (**a**) *TGMI* × VWCs map for 22 June 2012; (**b**) *TGMI* × VWCs map for 04 August 2013. Orange and red color indicates low values of *TGMI* × VWCs (low moisture), while green color indicates high values of *TGMI* × VWCs (high moisture). Urban areas, water bodies, clouds and cloud shadows are masked in black.

Figure 12a,b presents maps of VWC (*i.e.*, *TGMI* × VWCs) constructed for the study region from satellite data obtained on 22 June 2012 and 4 August 2013. Figure 12a was constructed from Landsat-7 multispectral image data, while Figure 12b was constructed from Landsat-8 multispectral image data. In the figures, *TGMI* × VWCs is color-coded to emphasize its variation across the landscape, and non-agricultural features (urban areas, water bodies), clouds, and cloud shadows have been masked as black. The information provided by *TGMI* is consistent with the known soil moisture conditions across the region. For the 22 June image (Figure 12a), *TGMI* exhibits greater spatial variation. The reason for this variation is that 2012 was a dry year so there are bigger differences between irrigated and non-irrigated fields. In the 04 August 2013 image (Figure 12b), *TGMI* is high in most areas (green color) with and little spatial variation. This is because of heavy rainfall few days before image acquisition. Image products such as these could be useful in monitoring regional soil moisture or drought conditions, and could provide input or calibration information for running models of crop growth and yield.

## 4. Conclusions

The results of this study suggest that *TGMI* is effective in estimating soil volumetric water content of agricultural fields under a variety of irrigation conditions ranging from fully irrigated to dryland. In this approach, *TGMI* can be evaluated on a pixel-by-pixel basis using raw image DC data without the need for conversion or calibration. Using measurements of volumetric soil water content obtained from 18 agricultural fields in the Texas High plains over 2 years, statistical analysis showed that *TGMI* × VWCs was closely related to soil moisture (R^2^ = 0.73, RMSE = 0.05). *TGMI* was used to construct maps showing the spatial distribution of soil moisture conditions over an agricultural region in which patterns of high and low *TGMI* were consistent with what would be expected from known crop management practices. Changes in the spatial distribution of *TGMI* over time were consistent with changes in irrigation in the region. Additional testing with measured soil moisture data will help assess the overall accuracy of this approach in estimating soil moisture, and identify its possible limitations. *TGMI* appears to be a potentially useful indicator of soil moisture that could find practical use in a range of applications, such as regional water resource monitoring and irrigation scheduling.

## Figures and Tables

**Table1.** Landsat image acquisitions used in this study.

**Year**	**Acquisition Date**		**Image Number**

	**Landsat-7**	**Landsat-8**	
2013	29 September	4 August	1
		5 September	2
		21 September	3
		-	4
		7 October	5

2012	22 June	-	6
	9 August	-	7
	25 August	-	8
	10 September	-	9
	26 September	-	10

## References

[1] Sobrino B., Franch C., Mattar J.C. (2012). A method to estimate soil moisture from airborne hyperspectral scanner (AHS) and aster data: Application to SEN2FLEX and SEN3EXP campaigns. Remote Sens. Environ.

[2] Cobari C., Sobrino J.A., Mancini M., Hidalgo V. (2010). Land surface temperature representativeness in a heterogeneous area through a distributed energy-water balance model and remote sensing data. Hydrol. Earth Syst. Sci.

[3] Wang L., Qu J.J., Zhang S., Hao X., Dasgupta S. (2007). Soil moisture estimation using MODIS and ground measurements in eastern China. Int. J. Remote Sens.

[4] Brocca L., Melone F., Moramarco T., Morbidelli R. (2010). Spatial-temporal variability of soil moisture and its estimation across scales. Water Resour. Res.

[5] Miralles W., Crow M. (2010). Estimating spatial sampling errors in coarse-scale soil moisture estimates derived from point-scale observations. J. Hydrometeorol.

[6] Wilson D., Western A.W. (2003). Spatial distribution of soil moisture over 6 and 30 cm depth, Machurangi river catchment, New Zealand. J. Hydrol.

[7] Piles M., Camps A., Vall-llossera M., Corbella I., Panciera R., Rudiger C., Kerr Y.H., Walker J. (2011). Downscaling SMOS-derived soil moisture using MODIS visible/infrared data. IEEE Trans. Geosci. Remote Sens.

[8] Jackson T.J., Cosh M.H., Bindlish R., Starks P.J., Bosch D.D., Seyfried M., Goodrich D.C., Moran M.S., Du J. (2010). Validation of advanced microwave scanning radiometer soil moisture products. IEEE Trans. Geosci. Remote Sens.

[9] Barrett J.E., Gooseff M.N., Takacs-vesbach C. (2009). Spatial variation in soil active-layer geochemistry across hydrologic margins in polar desert ecosystems. Hydrol. Earth Syst. Sci.

[10] Narayan U., Lakshmi V., Njoku E. (2004). Retrieval of soil moisture from passive and active L/S Band (PALS) observations during soil moisture experiment in 2002 (SMEX2002). Remote Sens. Environ.

[11] Sandholt I., Rasmussen K., Andersen J. (2002). A simple interpretation of the surface temperature/vegetation index space for assessment of soil moisture status. Remote Sens. Environ.

[12] Jiang L., Islam S.A. (2001). Estimation of surface evaporation map over Southern Great Plains using remote sensing data. Water Resour. Res.

[13] Engman E.T., Chauhan N. (1995). Status of microwave soil moisture measurements with remote-sensing. Remote Sens. Environ.

[14] Moran M.S., Clarke T.R., Inoue Y., Vidal A. (1994). Estimating crop water deficit using the relation between surface-air temperature and spectral vegetation index. Remote Sens. Environ.

[15] Dubois P.C., van Zyl J., Engman E.T. (1995). Measuring soil moisture with imaging radars. IEEE Trans. Geosci. Remote Sens.

[16] Goward S.N., Xue Y., Czajkowski K.P. (2002). Evaluating land surface moisture conditions from the remotely sensed temperature/vegetation index measurements: An exploration with the simplified simple biosphere model. Remote Sens. Environ.

[17] Wagner W., Guido L., Helmut R. (1999). A method for estimating soil moisture from ERS scatterometer and soil data. Remote Sens. Environ.

[18] Soliman A., Heck R.J., Brenning A., Brown R., Miller S. (2013). Remote sensing of soil moisture in vineyards using airborn and ground-based thermal inertia data. Remote Sens.

[19] Draper C., Mahfouf J.F., Calvet J.C., Martin E., Wagner W. (2011). Assimilation of ASCAT near-surface soil moisture into the SIM hydrological model over France. Hydrol. Earth Syst. Sci.

[20] Stisen S., Sandholt I., Norgaard A., Fensholt R., Jensen K.H. (2008). Combining the triangle method with thermal inertia to estimate regional evapotranspiration—Applied to MSG SEVIRI data in the Senegal River basin. Remote Sens. Environ.

[21] Wang J.Q., Moran S., Qi J., Marsett R. (2004). Soil moisture estimation in a semiarid rangeland using ERS-2 and TM imagery. Remote Sens. Environ.

[22] Carlson T.N., Capehart W.J., Gilies R.R. (1995). A new look at the simplified method for remote sensing of daily evapotranspiration. Remote Sens. Environ.

[23] Heilman J.L., Kanemasu E.T., Rosenberg N.J., Blad B.L. (1976). Thermal scanner measurement of canopy temperatures to estimate evapotranspiration. Remote Sens. Environ.

[24] Jackson R.D., Idso D.B., Reginato R.J., Pinter P.J. (1981). Canopy temperature as a crop water stress indicator. Water Resour. Res.

[25] Zhang D., Tang R., Zhao W., Tang B., Wu H., Shao K., Li Z.L. (2014). Surface Soil Water Content Estimation from Thermal Remote Sensing based on the Temporal Variation of Land Surface Temperature. Remote Sens.

[26] Carlson T.N., Gillies R.R., Perry E.M. (1994). A method to make use of thermal infrared temperature and NDVI measurements to infer surface soil water content and fractional vegetation cover. Remote Sens. Rev.

[27] Price J.C. (1990). Using spatial context in satellite data to infer regional scale evapotranspiration. IEEE Trans. Geosci. Remote Sens.

[28] Price J.C. (1980). The potential of remotely sensed thermal infrared data to infer surface soil moisture and evaporation. Water Resour. Res.

[29] Mallick K., Bhattacharya B.K., Patel N.K. (2009). Estimating volumetric surface moisture content for cropped soils using a soil wetness index based on surface temperature and NDVI. Agricul. For. Meteorol.

[30] Gillies R.R., Carlson T.N., Cui J., Kustas W.P., Humes K.S. (1997). Verification of the “triangle” method for obtaining surface soil water content and energy fluxes from remote measurements of the Normalized Difference Vegetation Index NDVI and surface radiant temperature. Int. J. Remote Sens.

[31] Nemani R.R., Pierce L., Running S.W., Goward S. (1993). Developing satellite-derived estimates of surface moisture status. J. Appl. Meteorol.

[32] Carlson T.N. (2007). An overview of the “triangle method” for estimating surface evapotranspiration and soil moisture from satellite imagery. Sensors.

[33] Petropoulos G., Carlson T.N., Wooster M.J., Islam S. (2009). A review of Ts/VI remote sensing based methods for the retrieval of land surface energy fluxes and soil surface moisture. Prog. Phys. Geogr.

[34] Friedl M.A., Davis F.W. (1994). Sources of variation in radioactive surface temperature over a tall grass prairie. Remote Sens. Environ.

[35] Gutman G., Ignatov A. (1998). The Derivation of the Green Vegetation Fraction from NOAA/AVHRR Data for Use in Numerical Weather Prediction Models. Int. J. Remote Sens.

[36] Maas S.J., Rajan N. (2008). Estimating ground cover of field crops using medium-resolution multispectral satellite imagery. Agron. J.

[37] Wang L.L. (2009). Satellite remote sensing applications for surface soil moisture monitoring: A review. Front. Earth Sci.

[38] Nelson J.R., Lascano R.J., Booker J.D., Zartman R.E., Goebel T.S. (2013). Evaluation of the Precision Agricultural Landscape Modeling System (PALMS) in the Semiarid Texas Southern High Plains. J. Soil Sci.

[39] USGS Science for a Changing World. http://earthexplorer.usgs.gov/.

[40] Landsat Processing Details. http://www.landsat.usgs.gov/Landsat_Processing_Details.php.

[41] Richardson A.J., Wiegand C.L. (1977). Distinguishing vegetation from soil background. Photogramm. Eng. Remote Sens.

[42] Bastiaanssen W.G.M., Menenti M., Feddes R., Holtslag A.M. (1998). Remote sensing surface energy balance algorithm for land (SEBAL). 1. Formulation. J. Hydrol.

